# Differential Gene Sets Profiling in Gram-Negative and Gram-Positive Sepsis

**DOI:** 10.3389/fcimb.2022.801232

**Published:** 2022-02-09

**Authors:** Qingliang Wang, Xiaojie Li, Wenting Tang, Xiaoling Guan, Zhiyong Xiong, Yong Zhu, Jiao Gong, Bo Hu

**Affiliations:** ^1^ Department of General Surgery, Third Affiliated Hospital of Sun Yat-sen University, Guangzhou, China; ^2^ Department of Laboratory Medicine, Third Affiliated Hospital of Sun Yat-sen University, Guangzhou, China; ^3^ Department of Molecular Diagnostics, Sun Yat-sen University Cancer Center, Guangzhou, China; ^4^ Department of General Surgery, The Fourth Affiliated Hospital of Anhui Medical University, Hefei, China

**Keywords:** sepsis, gene sets, Gram-positive, Gram-negative, microarray analysis

## Abstract

**Background:**

The host response to bacterial sepsis is reported to be nonspecific regardless of the causative pathogen. However, newer paradigms indicated that the host response of Gram-negative sepsis may be different from Gram-positive sepsis, and the difference has not been clearly clarified. The current study aimed to explore the difference by identifying the differential gene sets using the genome-wide technique.

**Methods:**

The training dataset GSE6535 and the validation dataset GSE13015 were used for bioinformatics analysis. The distinct gene sets of sepsis with different infections were screened using gene set variation analysis (GSVA) and gene set enrichment analysis (GSEA). The intersection gene sets based on the two algorithms were confirmed through Venn analysis. Finally, the common gene sets between GSE6535 and GSE13015 were determined by GSEA.

**Results:**

Two immunological gene sets in GSE6535 were identified based on GSVA, which could be used to discriminate sepsis caused by Gram-positive, Gram-negative, or mixed infection. A total of 19 gene sets were obtained in GSE6535 through Venn analysis based on GSVA and GSEA, which revealed the heterogeneity of Gram-negative and Gram-positive sepsis at the molecular level. The result was also verified by analysis of the validation set GSE13015, and 40 common differential gene sets were identified between dataset GSE13015 and dataset GSE6535 by GSEA.

**Conclusions:**

The identified differential gene sets indicated that host response may differ dramatically depending on the inciting organism. The findings offer new insight to investigate the pathophysiology of bacterial sepsis.

## Introduction

Sepsis is a potentially life-threatening condition caused mainly by bacterial infection, with high morbidity and mortality. It is now defined as infection accompanied by organ dysfunction resulting from dysregulated host responses (Singer et al., 2016). The early phase of sepsis is characterized by systemic excessive inflammation followed by a prolonged period of sepsis-induced immunosuppression (Delano and Ward, 2016). However, the pathophysiological mechanisms and host responses to sepsis have not been clearly elucidated, which hindered the development of new therapeutic approaches.

Although organs damaged by Gram-positive sepsis are clinically no different from Gram-negative sepsis, there is increasing evidence that differences exist in the host response (Li et al., 2017). The initiating factor of Gram-negative bacterial sepsis is endotoxin, while Gram-positive bacterial sepsis relies on the production of exotoxin (Ramachandran, 2014). Gram-negative sepsis differs from Gram-positive sepsis in that the organisms often arise from enteric or genitourinary sources rather than skin, wounds, and catheter sites (Martin, 2012). In addition, Gram-positive bacteria require a highly orchestrated host response, with intracellular killing by neutrophils and macrophages. This is different for Gram-negative pathogens, which may be readily killed in the extracellular space by antibody and complement (Van Amersfoort et al., 2003). It is gradually realized that the major difference between Gram-positive and Gram-negative sepsis is the way in which they initiate disease. Thus, exploring the difference in host response between Gram-negative and Gram-positive sepsis becomes increasingly important.

Microarray technology provides a powerful tool to examine genome-wide expression profiles. Although a great deal of information has become available for the molecular signature of sepsis (Chinnaiyan et al., 2001; Pop-Began et al., 2014; Lu et al., 2018), few reports have compared the difference between Gram-negative and Gram-positive sepsis. After analysis of the gene expression profiling of circulating neutrophils, Tang et al. verified that there was no difference in the expression profile. Gram-positive and Gram-negative sepsis share a common host response at a transcriptome level (Tang et al., 2008). However, the plasma IL-1β, IL-6, and IL-18 concentrations were significantly higher in Gram-positive sepsis patients even though the host inflammatory responses to Gram-negative and Gram-positive stimuli share some common response elements (Feezor et al., 2003).

The different mechanisms of sepsis caused by Gram−positive and Gram−negative bacteria were also illustrated previously (Giamarellos-Bourboulis et al., 2011; Mahabeleshwar et al., 2012; Kager et al., 2013). It was also reported that NADH: ubiquinone oxidoreductase subunit B2 (NDUFB2), NADH: ubiquinone oxidoreductase subunit B8 (NDUFB8), and ubiquinol−cytochrome c reductase hinge protein (UQCRH) may be associated with Gram−negative bacterial sepsis, while large tumor suppressor kinase 2 (LATS2) may contribute to the progression of Gram−positive bacterial sepsis (Li et al., 2017). Since sepsis was an overwhelming inflammatory response, it is really difficulty to distinguish the difference at the molecular level just with several differentially expressed genes. To further elucidate the effect of sepsis on host response, we undertook gene sets comparison analysis based on gene set variation analysis (GSVA) and gene set enrichment analysis (GSEA) in this study. By screening differentially expressed gene sets, we want to provide a novel approach to gain important biological insights into the host response of sepsis.

## Methods

### Microarray Data

The training dataset GSE6535 (Tang et al., 2008) and validation dataset GSE13015 (Pankla et al., 2009) were obtained from the Gene Expression Omnibus database (www.ncbi.nlm.nih.gov/geo). The original study was approved by the ethics committee of each institution, and written informed consent was provided by the patients or their families. There were totally 72 critically ill patients in GSE6535, 17 of whom were served as control. Based on the results of clinical features and microbiological culture, 18 patients were diagnosed as Gram−positive sepsis, 25 were confirmed as Gram-negative sepsis, while 12 were identified as mixed sepsis. The type of infection for mixed sepsis was pneumonia (four cases), intra-abdominal infection (six cases), urinary tract infection (one case), and meningitis (one case). There were nine cases of pneumonia, one case of intra-abdominal infection, and eight cases of other infections for Gram-positive sepsis, while five cases of pneumonia, one case of intra-abdominal infection, eight cases of urinary tract infection, four cases of meningitis, and seven cases of other infections for Gram-negative sepsis. The neutrophil RNA was isolated within 24 h of admission and microarray experiments were then performed. Whole blood of 63 patients with sepsis was used to generate genome-wide transcriptional profiles in GSE13015. All patients were diagnosed as sepsis based on blood culture, including 43 patients with Gram-negative bacteria (mainly *Burkholderia pseudomallei*), 3 patients with fungi, and 17 patients with Gram-positive sepsis. Owing to the biased data of Gram-negative sepsis, we only randomly selected four cases of *B. pseudomallei* for further analysis. The analyzed microbiology data in this study were also summarized (**Table 1**).

**Table 1:** Microbiology data analyzed in this study.

GSE6535			GSE13015	
Gram-positive (18)	Gram-negative (25)	Mixed (12)	Gram-positive (17)	Gram-negative (15)
*Streptococcus* (8)	*Escherichia* (11)	Mixed anaerobes (6)	Coagulase-negative staphylococcus (6)	*Escherichia* (6)
*Staphylococcus* (5)	*Pseudomonas* (4)	*Escherichia* (4)	*Corynebacterium* spp. (3)	*B. pseudomallei* (4)
MRSA (3)	*Neisseria* (3)	MRSA (4)	*S. aureus* (2)	*K. pneumoniae* (1)
*Enterococcus* (1)	*Klebsiella* (1)	*Enterococcus* (4)	*Streptococcus* non-group A or B (1)	*A. baumannii* (1)
*Listeria* (1)	*Citrobacter* (1)	*Klebsiella* (4)	*Staphylococcus aureus* (1)	*Salmonella* serotype B (1)
	*Enterobacter* (1)	*Pseudomonas* (3)	*Enterococcus* spp. (1)	*Salmonella* spp. (1)
	*Proteus* (1)	*Streptococcus* (3)	*S. pneumoniae* (1)	*A. hydrophila* (1)
	*Bacteroides* (1)	*Stenotrophomonas* (2)	*Enterococcus* spp. (1)	
	*Haemophilus* (1)	*Nocardia* (1)	*E. faecium* (1)	
	*Serratia* (1)	*Haemophilus* (1)		
		*Staphylococcus* (1)		

### Gene Set Variation Analysis

GSVA was applied to assess individual samples using a non-parametric approach in dataset GSE6535. Probe IDs were first converted into their corresponding gene symbols. GSVA package in R platform (4.0.3) was used to calculate the enrichment score of the pathways in each sample, while *p <*0.05 was considered statistically significant. The results were then visualized in a heatmap, generated by the ComplexHeatmap package in R. The reference gene sets were the Hallmark gene sets, C2 gene sets, and C7 gene sets owing to their close relationship to sepsis. Subsequently, the common gene sets between Gram−positive and Gram−negative samples, Gram−positive and mixed samples, and Gram−negative and mixed samples were identified with the Venn Diagram in R.

### Protein–Protein Interaction Network Analysis

Protein–protein interaction (PPI) network was analyzed with the online database Search Tool for the Retrieval of Interacting Genes (STRING 11.0, https://string-db.org). The distinct gene-sets-encoded proteins were employed to build the PPI network with the default threshold value (a combined score ≥0.4). Then, the PPI network was constructed by means of Cytoscape software (version 3.8.0), and the plug-in of Molecular Complex Detection (MCODE) and cytoHubba were applied for further analysis. The criteria for selection was that MCODE scores >5.

### Gene Set Enrichment Analysis

GSEA is a computational method for assessing whether a set of genes defined by *a priori* show statistical significance between two biological states. It was used to explore the differential gene sets between Gram-negative and Gram-positive sepsis in dataset GSE6535 and GSE13015. The annotated gene sets related to sepsis, “C2, curated gene sets”, “C7, gene immunologic signature gene sets”, and “Hallmark gene sets”, downloaded from the Molecular Signature Database (MSigDB), were considered as the reference gene sets. The number of permutations was 1,000, and other parameters were set to default. A significant difference at *p*-value <0.05 was defined as the cutoff criteria after 1,000-time permutations.

### GO and KEGG Enrichment Analysis

Gene Ontology (GO) and Kyoto encyclopedia of Genes and Genomes (KEGG) were used to elucidate the potential gene functional annotation and pathway enrichment. Both GO and KEGG analyses were performed by R package “cluster Profiler”, and adjusted *p*-value <0.05 were regarded as statistically significant. GO analysis was comprised of biological process (BP), cellular component (CC), and molecular function (MF) and described the facilities of genes in three distinct biological aspects. Enrichment maps visualizing the results were drawn by R Software and Bioconductor (http://bioconductor.org/).

## Results

### Identify the Distinct Gene Sets Based on GSVA

**Figure 1:**
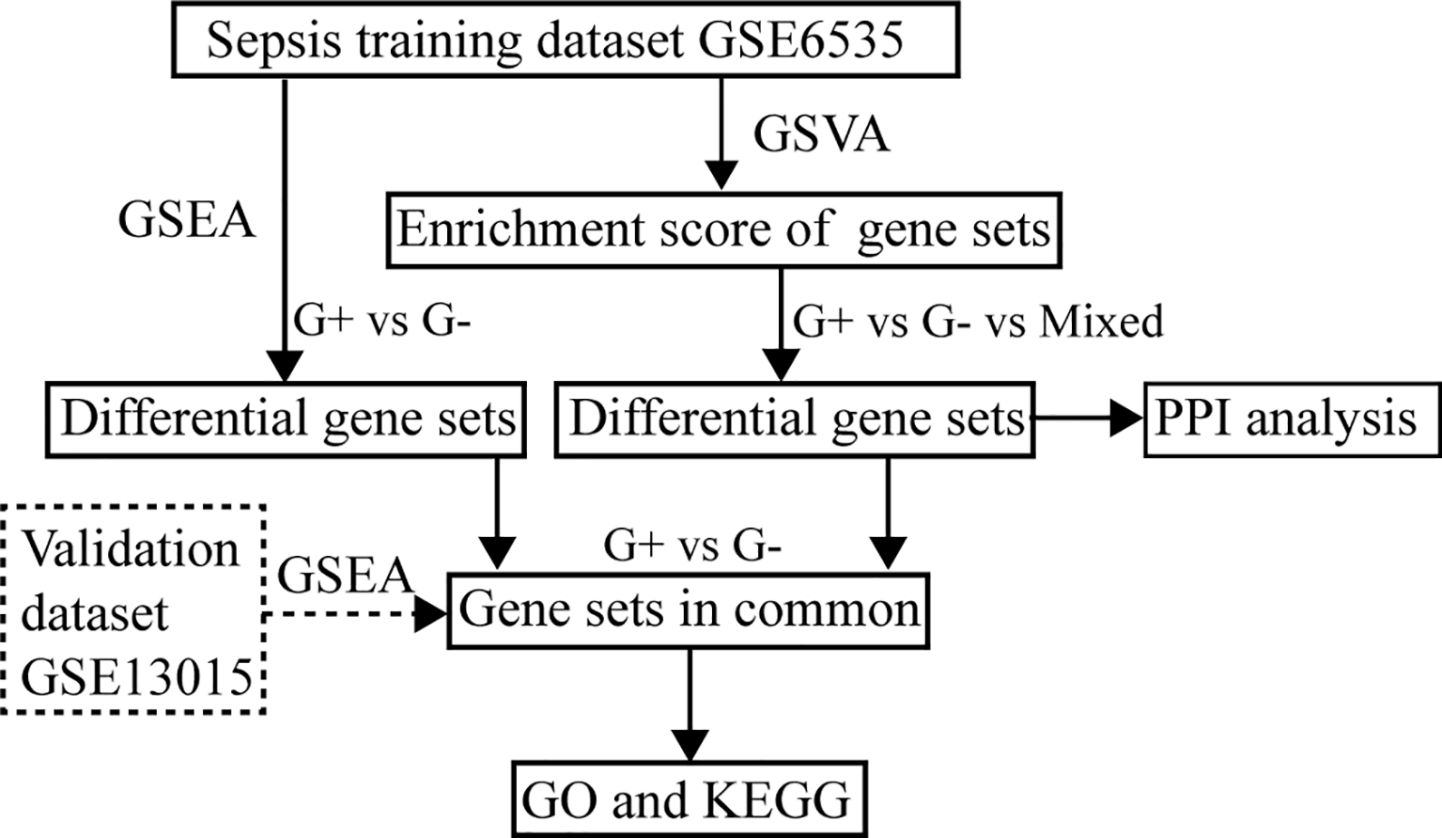
Analysis workflow of this study.

**Figure 2:**
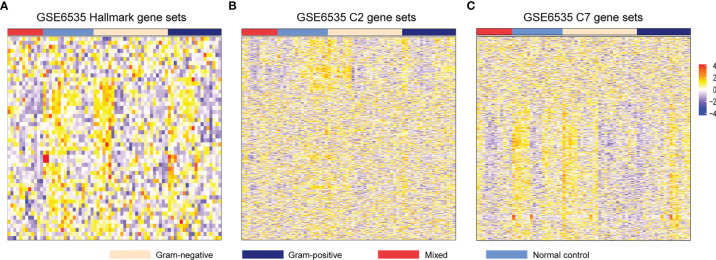
Heatmap of enrichment score of **(A)** Hallmark gene sets, **(B)** C2 gene sets, and **(C)** C7 gene sets in patients with Gram−positive sepsis, Gram-negative sepsis, mixed sepsis, and normal control. The rows in the heatmap indicate the expression values of each gene set, and the columns indicate the 72 samples examined in dataset GSE6535.

**Figure 3:**
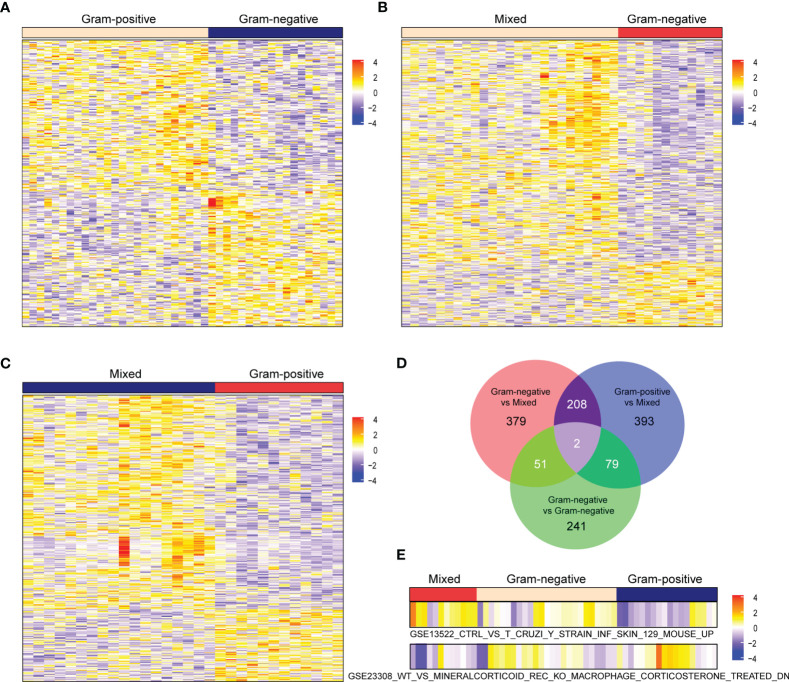
Heatmap of differential gene sets between **(A)** Gram-positive and Gram-negative sepsis, **(B)** mixed sepsis versus Gram-negative sepsis, and **(C)** mixed sepsis versus Gram-positive sepsis. Venn diagram of **(D)** differential gene sets across various infection types and **(E)** the identified two distinct gene sets.

The flowchart of this study is illustrated in **Figure 1**. All patients in GSE6535 were grouped according to the infection status and analyzed by GSVA. The variation in the activity for gene sets was estimated, and the matrix containing enrichment scores was depicted in a heatmap (**Figure 2**). Next, the enrichment score (ES) of gene sets between Gram-positive sepsis patients and Gram-negative sepsis patients was compared. A total of 373 differential gene sets were confirmed. The heatmap showed that the ES patterns may distinguish Gram-positive sepsis patients from Gram-negative sepsis patients easily (**Figure 3A**). In addition, we also screened 640 differential gene sets between Gram-negative sepsis patients and mixed infection patients and 682 differential gene sets between Gram-positive sepsis patients and mixed infection patients, which were also displayed in the heatmap (**Figures 3B, C**). After intersection analysis, two distinct immunologic gene sets, namely, “GSE13522_CTRL_VS_T_CRUZI_Y_STRAIN_INF_SKIN_129_MOUSE_UP” and “GSE23308_WT_VS_MINERALCORTICOID_REC_KO_MACROPHAGE_CORTICOSTERONE_TREATED_DN” were identified (**Figure 3D**). The detailed expression of each infected patient was also described in the heatmap, in which Gram-positive sepsis patients exhibit the relatively highest expression in gene set “GSE23308_WT_VS_MINERALCORTICOID_REC_KO_MACROPHAGE_CORTICOSTERONE_TREATED_DN” and the lowest expression in gene set “GSE13522_CTRL_VS_T_CRUZI_Y_STRAIN_INF_SKIN_129_MOUSE_UP” (**Figure 3E**). The complete gene list of the two gene sets is also shown (**Supplementary Table S1**).

### PPI Network Construction, Module Analysis, and Hub Genes Identification

**Figure 4:**
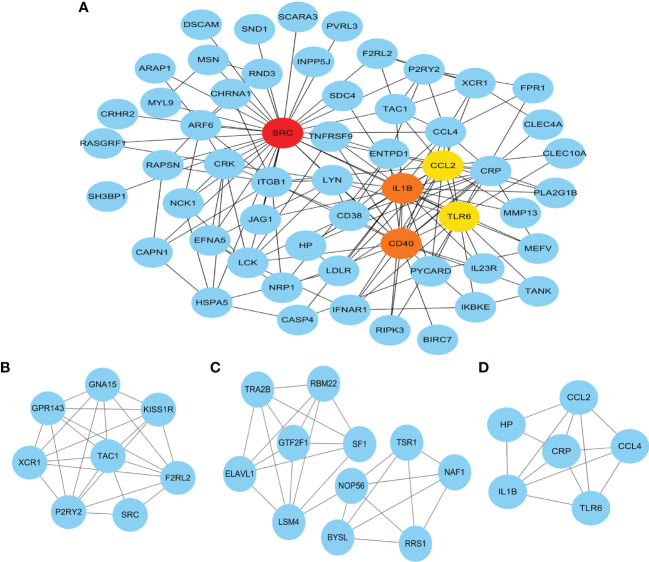
Protein–protein interaction network of the two distinct gene sets, namely, **(A)** the top 5 hub genes and **(B–D)** the top 3 clusters.

Next, the PPI network of the two distinct gene sets (335 genes) was constructed from STRING. Based on the information of the public database, a total of 242 nodes and 479 protein pairs were obtained, while the isolated genes without interaction were removed. To further investigate the hub genes, the plug-in app “cytoHubba” was used to parse the network, and the top 5 hub genes were identified according to the “Degree” algorithm (**Figure 4A**), including SRC (degree = 33), IL1B (degree = 20), CD40 (degree = 20), TLR6 (degree = 16), and CCL2 (degree = 16). After that, the module analysis was performed by MCODE, and three modules were screened. Module 1 was the most significant module, located in the center of the entire PPI network, including 8 genes and 24 edges (**Figure 4B**). Modules 2 and 3 had 11 nodes (**Figure 4C**) and 6 nodes (**Figure 4D**), respectively, containing several hub genes such as IL1B, TLR6, and CCL2 (**Figure 4D**).

### Screening Differential Gene Sets With GSEA and GSVA

**Figure 5:**
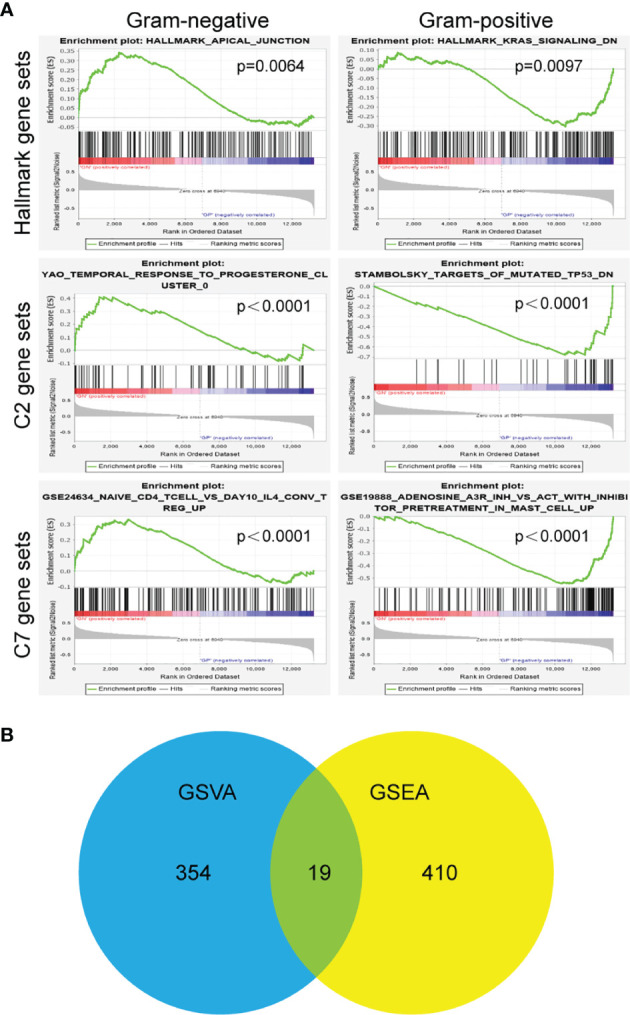
Gene set enrichment analysis for dataset GSE6535. **(A)** Representative images of annotated gene sets with p value. **(B)** Venn diagram of the common differential gene sets between Gram-negative and Gram-positive sepsis.

To further elucidate the different pathway involved in Gram-positive and Gram-negative sepsis, GSEA was performed between the two groups in GSE6535. It evaluates the microarray data by performing unbiased global searches for genes that are coordinately regulated in the three predefined gene sets. The results showed a significant difference in enrichment. The analysis of the Hallmark gene sets revealed that there were four significantly enriched gene sets, namely, HALLMARK_APICAL_JUNCTION, HALLMARK_NOTCH_SIGNALING, HALLMARK_KRAS_SIGNALING_DN, and HALLMARK_INTERFERON_ALPHA_RESPONS. The enrichment of C2 indicated that there were 226 differential gene sets, while the enrichment of C7 showed 199 differential gene sets. The representative plots of each gene sets with the lowest *p*-value are shown in **Figure 5A**. After that, the intersection gene sets based on the two algorithms, GSVA and GSEA, were finally confirmed through Venn analysis (**Figure 5B**). A total of 19 gene sets were obtained (**Table 2**), most of which are related to immunity.

**Table 2:** The common differential gene sets between Gram-negative and Gram-positive sepsis based on GSVA and GSEA for dataset GSE6535.

Gene sets	Collections
MANNE_COVID19_NONICU_VS_HEALTHY_DONOR_PLATELETS_UP	C2
GSE19825_NAIVE_VS_IL2RALOW_DAY3_EFF_CD8_TCELL_UP	C7
GSE4142_PLASMA_CELL_VS_MEMORY_BCELL_DN	C7
GSE21546_UNSTIM_VS_ANTI_CD3_STIM_SAP1A_KO_AND_ELK1_KO_DP_THYMOCYTES_UP	C7
GSE45365_CD8A_DC_VS_CD11B_DC_IFNAR_KO_UP	C7
GSE1432_CTRL_VS_IFNG_24H_MICROGLIA_DN	C7
MIKKELSEN_MEF_LCP_WITH_H3K4ME3	C2
GSE34006_WT_VS_A2AR_KO_TREG_DN	C7
GSE40273_EOS_KO_VS_WT_TREG_DN	C7
GSE21927_SPLENIC_C26GM_TUMOROUS_VS_BONE_MARROW_MONOCYTES_UP	C7
REACTOME_RHO_GTPASES_ACTIVATE_WASPS_AND_WAVES	C2
GSE41176_UNSTIM_VS_ANTI_IGM_STIM_TAK1_KO_BCELL_6H_UP	C7
HUPER_BREAST_BASAL_VS_LUMINAL_UP	C2
GSE17721_CTRL_VS_LPS_1H_BMDC_UP	C7
GSE21360_NAIVE_VS_QUATERNARY_MEMORY_CD8_TCELL_DN	C7
GSE37533_PPARG1_FOXP3_VS_FOXP3_TRANSDUCED_CD4_TCELL_PIOGLITAZONE_TREATED_UP	C7
GRAESSMANN_RESPONSE_TO_MC_AND_SERUM_DEPRIVATION_UP	C2
GSE37534_UNTREATED_VS_PIOGLITAZONE_TREATED_CD4_TCELL_PPARG1_AND_FOXP3_TRASDUCED_DN	C7
GSE21546_WT_VS_SAP1A_KO_DP_THYMOCYTES_UP	C7

### GO and KEGG Enrichment Analysis

**Figure 6:**
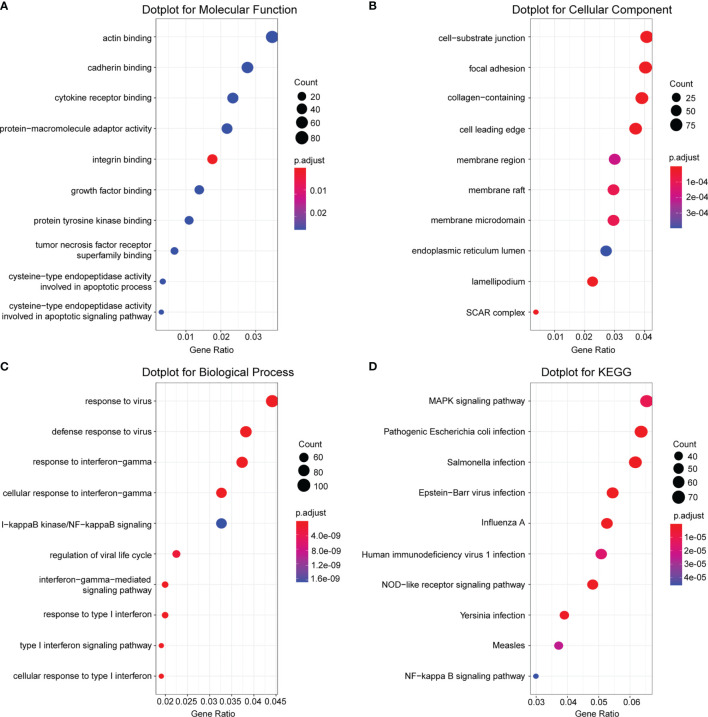
Gene Ontology (GO) and Kyoto Encyclopedia of Genes and Genomes (KEGG) pathway of the genes involved in the intersection gene sets. **(A)** Molecular function, **(B)** cellular component, and **(C)** biological process for GO analysis. **(D)** The top 10 of KEGG pathway enrichment.

To gain more biological insight into the screened gene sets, GO annotation and KEGG pathway enrichment analysis were conducted with the 19 gene sets. The top 10 enriched GO terms and KEGG pathways were identified and presented in **Figure 6**. GO analysis showed that the most enriched MF terms were actin binding, cadherin binding, cytokine receptor binding, and protein–macromolecule adaptor activity (**Figure 6A**). For GO CC analysis, the top 5 significantly enriched terms were cell–substrate junction, focal adhesion, collagen-containing extracellular matrix, cell leading edge, and membrane region (**Figure 6B**). In the BP, the genes were mainly enriched in response to virus, defense response to virus, response to interferon-gamma, cellular response to interferon-gamma, and nuclear factor kappa B (NF-κB) signaling (**Figure 6C**). KEGG pathway analysis demonstrated that genes were mainly enriched in mitogen-activated protein kinase (MAPK) signaling pathway, pathogenic *Escherichia coli* infection, *Salmonella* infection, *Epstein–Barr virus* infection, and *Influenza A* (**Figure 6D**).

### Differential Gene Sets Verification With GSE13015

The differential gene sets between Gram-positive and Gram-negative sepsis were further verified with dataset GSE13015. According to GSEA, there were 9 significantly enriched gene sets in the Hallmark gene sets, 750 gene sets in C2 collections, and 819 gene sets in C7 collections. The further analysis showed that there were 40 common differential gene sets based on GSEA between dataset GSE13015 and dataset GSE6535 (**Table 3**). In addition, the results also verified our conclusion in GSE6535, two additional intersection gene sets were confirmed after Venn analysis with the 19 gene sets, REACTOME_RHO_GTPASES_ACTIVATE_WASPS_AND_WAVES and MIKKELSEN_MEF_LCP_WITH_H3K4ME3.

**Table 3:** The common differential gene sets between GSE6535 and GSE13015 based on gene set enrichment analysis.

Gene sets	Collections	Gene sets	Collections
HALLMARK_APICAL_JUNCTION	H	GSE18791_UNSTIM_VS_NEWCATSLE_VIRUS_DC_2H_DN	C7
SCHAEFFER_PROSTATE_DEVELOPMENT_12HR_UP	C2	GSE17721_CTRL_VS_LPS_6H_BMDC_DN	C7
SIG_INSULIN_RECEPTOR_PATHWAY_IN_CARDIAC_MYOCYTES	C2	GSE17721_CTRL_VS_GARDIQUIMOD_2H_BMDC_DN	C7
BERENJENO_ROCK_SIGNALING_NOT_VIA_RHOA_DN	C2	GSE20500_CTRL_VS_RETINOIC_ACID_TREATED_CD4_TCELL_DN	C7
WP_CELL_MIGRATION_AND_INVASION_THROUGH_P75NTR	C2	GSE35685_CD34POS_CD38NEG_VS_CD34POS_CD10NEG_CD62LPOS_BONE_MARROW_DN	C7
KEGG_AXON_GUIDANCE	C2	GSE15930_NAIVE_VS_24H_IN_VITRO_STIM_INFAB_CD8_TCELL_UP	C7
TIEN_INTESTINE_PROBIOTICS_2HR_UP	C2	GSE7460_WT_VS_FOXP3_HET_ACT_TCONV_UP	C7
WP_G_PROTEIN_SIGNALING_PATHWAYS	C2	GSE15930_NAIVE_VS_24H_IN_VITRO_STIM_CD8_TCELL_UP	C7
REACTOME_RHO_GTPASES_ACTIVATE_WASPS_AND_WAVES	C2	GSE17721_POLYIC_VS_GARDIQUIMOD_8H_BMDC_UP	C7
LEONARD_HYPOXIA	C2	GSE6269_FLU_VS_E_COLI_INF_PBMC_UP	C7
MIKKELSEN_MCV6_LCP_WITH_H3K4ME3	C2	GSE9037_CTRL_VS_LPS_1H_STIM_IRAK4_KO_BMDM_DN	C7
WP_TOLLLIKE_RECEPTOR_SIGNALING_RELATED_TO_MYD88	C2	GSE21670_STAT3_KO_VS_WT_CD4_TCELL_UP	C7
MIKKELSEN_MEF_LCP_WITH_H3K4ME3	C2	GSE7831_UNSTIM_VS_INFLUENZA_STIM_PDC_4H_UP	C7
WP_FIBRIN_COMPLEMENT_RECEPTOR_3_SIGNALING_PATHWAY	C2	GSE46242_TH1_VS_ANERGIC_TH1_CD4_TCELL_UP	C7
REACTOME_MUSCLE_CONTRACTION	C2	GSE24634_IL4_VS_CTRL_TREATED_NAIVE_CD4_TCELL_DAY5_UP	C7
WP_EICOSANOID_METABOLISM_VIA_LIPO_OXYGENASES_LOX	C2	GSE21360_NAIVE_VS_SECONDARY_MEMORY_CD8_TCELL_DN	C7
REACTOME_CARDIAC_CONDUCTION	C2	GSE1460_INTRATHYMIC_T_PROGENITOR_VS_DP_THYMOCYTE_DN	C7
BIDUS_METASTASIS_DN	C2	GSE360_DC_VS_MAC_B_MALAYI_HIGH_DOSE_DN	C7
WP_TLR4_SIGNALING_AND_TOLERANCE	C2	GSE37534_UNTREATED_VS_GW1929_TREATED_CD4_TCELL_PPARG1_AND_FOXP3_TRASDUCED_UP	C7
MEBARKI_HCC_PROGENITOR_FZD8CRD_DN	C2	GSE22935_UNSTIM_VS_24H_MBOVIS_BCG_STIM_MYD88_KO_MACROPHAGE_DN	C7

## Discussion

In the present study, the host response to different invading pathogens was assessed using gene expression patterns. The results from the training dataset revealed that the expression profiling of neutrophils could reliably distinguish the molecular difference. Exploring the potential difference in sepsis is essential to further understand the mechanism. GSVA provides increased power to detect subtle pathway activity changes in an unsupervised manner (Hanzelmann et al., 2013). After GSVA enrichment and intersection analysis, two distinct immunological gene sets were confirmed, which can be used to discriminate the different types of sepsis. It also indicated that the host immune system is activated even in the early stage of sepsis, rather than at the classic anti-inflammatory phase (Tang et al., 2010).

The functional interaction between proteins was also analyzed in the current study. Three densely connected regions and several hub genes were identified, which revealed important biological insights into the host response mediated by neutrophils. SRC belongs to the protein tyrosine kinases (PTKs) family and plays a critical role in initiating the numerous intracellular signaling pathway that affects cell migration, adhesion, phagocytosis, cell cycle, and cell survival (Korade-Mirnics and Corey, 2000). It has been identified to be essential for the recruitment and activation of monocytes, macrophages, neutrophils, and other immune cells. It also plays a critical role in the regulation of vascular permeability and inflammatory responses in tissue cells (Okutani et al., 2006). Toll-like receptors (TLRs) play an essential role in pathogen recognition and activation of innate immunity. TLR6 acts in a heterodimer form with TLR2, which mediates cell response to Gram-positive bacterial components. TLR2 regulates important neutrophil functions, including adhesion, generation of reactive oxygen species, release of chemokines, and activation of major proinflammatory signaling pathways, such as NF-κB pathway (Andrews et al., 2013). IL1B is an important mediator of the inflammatory response and participates in a variety of cellular activities, including cell proliferation, differentiation, and apoptosis (Liu and Sun, 2019). CD40 is a receptor in antigen-presenting cells of the immune system and is essential for mediating a broad variety of immune and inflammatory responses (Michels et al., 2015). CCL2 is one of the key chemokines that regulate migration and infiltration of monocytes and macrophages (Carson et al., 2017).

Although the clinical manifestations of sepsis caused by Gram-negative and Gram-positive bacteria may appear similar, our study indicated that the host physiological response to these pathogens may behave differently due to the inciting organism. The findings were concordant with the results of Feezor et al., the host inflammatory responses to Gram-negative and Gram-positive stimuli not only share some common response elements but also exhibit distinct patterns of cytokine appearance and leukocyte gene expression (Feezor et al., 2003). It was also confirmed by genome-wide gene expression analysis of a mouse sepsis model after infusion of either live *Escherichia coli* or *Staphylococcus aureus* (Yu et al., 2004). The study of Li et al. also determined that there was no significant difference in the expression profile between Gram−positive and Gram−negative samples; however, several candidate genes may be biomarkers for distinguishing the different infections (Li et al., 2017). Unlike these reports, the current study mainly focuses on the differences in pathways or gene sets rather than a single gene because no single molecule can recapitulate the complex changes that occur in sepsis.

Gram-positive and Gram-negative bacteria activate different receptor pathways in the host, among which Toll-like receptors play a pivotal role (Elson et al., 2007). TLR4 is regarded as the major lipopolysaccharide receptor for Gram-negative bacteria (Branger et al., 2004), whereas cellular responses to components of Gram-positive bacteria are mainly mediated *via* TLR2 (Oliveira-Nascimento et al., 2012). Individual TLRs differentially recruit specific adaptor molecules, such as MyD88, TRIF, TIRAP/MAL, or TRAM, leading to the activation of NF-κB and MAP kinases pathways (Kawasaki and Kawai, 2014). The results were also confirmed in our study after KEGG analysis; the genes were mainly enriched in MAPK signaling pathway. It was also reported that combined signaling of TLR2 and CD137 augments antibacterial activities of neutrophils while that of TLR4-CD137 diminishes them (Nguyen et al., 2013). Gram-negative and Gram-positive bacteria do not trigger monocyte activation through similar pathways. Lipopolysaccharide but not *S. aureus* Cowan used CD14 internalization to induce cellular activation, resulting in p38 MAP kinase and ERK kinase activation pathways (Takeuchi et al., 1999). Besides that, host-response pathway correlated metabolites could be used to distinguish between bacterial- and host-induced metabolic changes (Hoerr et al., 2012).

According to the sepsis guidelines, empiric antimicrobial therapy was recommended before obtaining blood cultures (Dellinger et al., 2013). However, the increasing antibiotic resistance requires novel approaches for early identification of the causative microorganism (Najeeb et al., 2012). After analyzing the plasma free circulating DNA from sepsis patients, Grumaz et al. developed an alternative diagnostic platform to identify infectious microorganisms in roughly 30 h by next-generation sequencing (Grumaz et al., 2016). Recently, the focus for accurate and rapid diagnosis has moved from single disease-specific markers to bioprofiles or biosignatures comprising a well-defined set of reliable molecular indicators using platforms such as proteomics (Vincent et al., 2010) transcriptomics (Zhang et al., 2010), genomics (Parida and Kaufmann, 2010), and metabolomics (Claus et al., 2010). In this current study, besides the 19 gene sets identified in GSE6535 based on GSVA and GSEA, we also identified 40 gene sets based on GSEA in the two datasets, of which 20 gene sets were immunological signature gene sets. Based on our results, the differential gene sets between Gram-negative and Gram-positive sepsis could be further explored for diagnosis purpose with the immunoassay.

The data used in the training dataset were obtained from neutrophils collected within 24 h. We chose neutrophils instead of other leukocytes because neutrophils are crucial components of an early host’s innate immune response (Kovach and Standiford, 2012). Experimental conditions were similar for all patients to minimize the difference between individual patients. Nonetheless, there are some limitations. The findings were based on a microarray dataset from a single institution with small sample size. Although similar results were obtained in the validation dataset, a large sample from multiple centers is needed to further verify our results. On the other hand, gene expression profiles are known to change rapidly in the early stages of sepsis (Maslove and Wong, 2014). Thus, the timing of microarray analysis should also be considered to consolidate our results. In addition, specimens from different sources may affect the expression characteristics of the genome. In the validation dataset GSE13015, whole blood contains a mixed population of leukocytes, the proportion of which varies depending on the stage of sepsis and between individuals. However, the common gene sets in the two datasets also indicated the molecular difference between Gram-negative and Gram-positive sepsis.

In summary, our results highlight the heterogeneity of Gram-negative and Gram-positive sepsis at the molecular level. The screened differential gene set indicated that host response may differ dramatically depending on the inciting organism. The findings offer new insight to investigate the initiating mechanisms of sepsis and provide a potential method to identify the causative organism at the onset of sepsis.

## Data Availability Statement

The datasets presented in this study can be found in online repositories. The names of the repository/repositories and accession number(s) can be found in the article.

## Ethics Statement

The studies involving human participants were reviewed and approved by the ethics committee of each institution, and written informed consent was provided by the patients or their families. The patients/participants provided their written informed consent to participate in this study. Written informed consent was obtained from the individual(s) for the publication of any potentially identifiable images or data included in this article.

## Author Contributions

JG and BH conceived and developed the study and obtained funding for the study. QW and XL wrote the manuscript and prepared the figures. JG, XG, and ZX conducted the biostatistical analysis. YZ contributed to the data collection. All authors contributed to the article and approved the submitted version.

## Funding

This study was supported by the grant from the Science and Technology Program of Guangzhou, China (201903010039 and 202102010199), Basic and Clinical Cooperative Research Promotion Program of Anhui Medical University (2019xkjT029), Clinical Medicine Discipline Construction Project of Anhui Medical University(2020lcxk032), and Fundamental Research Funds for the Central Universities (20ykpy21).

## Conflict of Interest

The authors declare that the research was conducted in the absence of any commercial or financial relationships that could be construed as a potential conflict of interest.

## Publisher’s Note

All claims expressed in this article are solely those of the authors and do not necessarily represent those of their affiliated organizations, or those of the publisher, the editors and the reviewers. Any product that may be evaluated in this article, or claim that may be made by its manufacturer, is not guaranteed or endorsed by the publisher.
