# Supplementary material for: Differential Gene Sets Profiling in Gram-Negative and Gram-Positive Sepsis
Source: Front Cell Infect Microbiol. 2022 Feb 9;12:801232. doi: 10.3389/fcimb.2022.801232 (PMC8863667; doi:10.3389/fcimb.2022.801232)
Supplement: Supplementary file 1 [file Table_1.docx]

**Table S1** Gene list of the two distinct immunologic gene sets.

| **GSE13522_CTRL_VS_T_CRUZI_Y_STRAIN_INF_SKIN_129_MOUSE_UP** | | | | **GSE23308_WT_VS_MINERALCORTICOID_REC_KO_MACROPHAGE_CORTICOSTERONE_TREATED_DN** | | | | |
| --- | --- | --- | --- | --- | --- | --- | --- | --- |
| ABCA2 | GNA15 | NR6A1 | SYT5 | ACTR1A | DYNLL1 | KRCC1 | PGK1 | SLC6A3 |
| ABCA4 | GNPTG | NRN1L | TAAR9 | ALG9 | EFNA5 | LCK | PGK2 | SMIM30 |
| ABRA | GPR143 | NRP1 | TM4SF20 | ALX3 | ELAVL1 | LDLR | PGLYRP2 | SNF8 |
| ADORA2B | GPR82 | OC90 | TMEM45B | AP2S1 | EMID1 | LSM4 | PGP | SP2 |
| AIDA | GRIN2C | PANX3 | TNNI2 | APBA3 | EPRS1 | LYN | PHGDH | SPIDR |
| ALDH5A1 | HHEX | PARD6G | UBE2L3 | ARAP1 | ERO1A | MAP11 | PHLDA1 | SPRED1 |
| AMZ1 | HNMT | PCLO | UBQLN1 | ARF6 | ETF1 | MAPRE1 | PIN1 | ST3GAL1 |
| ARRDC4 | HPS6 | PGLYRP4 | WFDC5 | ARMH4 | ETV6 | MARCKSL1 | POFUT2 | STAG1 |
| ASPG | IGFN1 | PHEX | XCR1 | ARPC5 | FAM32A | MC3R | PRKG2 | STK17B |
| BIRC7 | IL23R | PKHD1 | ZAN | ARVCF | FMR1 | MED14 | PROSER1 | STRN4 |
| BMP8B | INPP5J | PLA2G1B | ZDHHC1 | ATG3 | FOXK2 | MED29 | PRPF38B | STX12 |
| CAPN1 | ITPKA | PLA2G4F | ZER1 | BLOC1S3 | FOXN2 | MEFV | PRSS42P | SUN1 |
| CASTOR2 | IZUMO1 | PLCD3 | ZFP28 | BYSL | FPR1 | MMP13 | PSMD7 | SUPT6H |
| CBLIF | KATNAL2 | PLIN5 | ZP3 | C12orf66 | GARS1 | MRPL45 | PSPC1 | TAC1 |
| CD40 | KCNK7 | PNPLA1 | ZSCAN29 | C5orf24 | GDPD1 | MRPL52 | PYCARD | TANK |
| CLEC10A | KCNMB4 | POMGNT2 |  | CA8 | GLIPR2 | MSN | RAB20 | TARS1 |
| CLEC4G | KIF13A | POU2F3 |  | CABP2 | GMFG | MT2A | RAMP2 | TLR6 |
| CNTN1 | KIF26B | PRR15L |  | CAMKK2 | GORASP2 | MTMR14 | RAN | TMEM116 |
| COL15A1 | KIF5C | PSMB11 |  | CAPZA1 | GTF2A1 | MTMR6 | RAP1GDS1 | TMEM119 |
| COL9A1 | KRT39 | RAPSN |  | CASP4 | GTF2F1 | MYO1H | RBM22 | TMEM63A |
| CPLX2 | LRRC63 | RARRES1 |  | CCDC115 | GUCA2B | NAB2 | RBPMS2 | TNFRSF9 |
| CRISPLD1 | LY6G6C | RASGRF1 |  | CCL2 | HEATR6 | NCK1 | RIPK3 | TNIP2 |
| CRP | MDGA1 | RBM20 |  | CCL4 | HOXB2 | NCOA5 | RLF | TNNT2 |
| CTU1 | MED11 | RBP7 |  | CD38 | HP | NDEL1 | RND3 | TOX4 |
| DPPA4 | MEP1B | RDH8 |  | CDC42SE1 | HSPA5 | NECAP2 | RNF14 | TPM3 |
| ENTPD1 | MIR194-2 | REPS1 |  | CDS2 | IARS1 | NECTIN3 | RNF149 | TRA2B |
| EPHA10 | MIR200B | RPRD1B |  | CHORDC1 | IDH3A | NEK6 | RRAD | TRAPPC13 |
| ERN2 | MIR491 | RRS1 |  | CHRNA1 | IDNK | NEUROG1 | RWDD4 | TRAPPC4 |
| ESAM | MIRLET7B | RSPH14 |  | CIAO2B | IFNAR1 | NOL12 | SAMD4B | TRPS1 |
| EZHIP | MORN3 | S100A11 |  | CLCN5 | IGFBP5 | NOP56 | SAV1 | TSR1 |
| F2RL2 | MSRB2 | S100A16 |  | CLEC4A | IGFBP6 | NUBP2 | SEC11A | TTC1 |
| FAM170B | MTARC1 | SCARA3 |  | CNOT7 | IKBKE | NUPR1 | SEC24B | VAPA |
| FAM222B | MYL9 | SDC4 |  | COMMD7 | IKZF4 | NXT1 | SECISBP2L | VSTM2A |
| FAM24A | NAF1 | SEMA6A |  | CPA2 | IL1B | ODF4 | SEMA3F | YARS1 |
| FAM43A | NBL1 | SND1 |  | CRHR2 | IST1 | OPRL1 | SEPTIN11 | ZBTB7B |
| FASN | NDUFS5 | SOHLH1 |  | CRK | ITGB1 | P2RY2 | SEPTIN7 | ZFAND3 |
| FBXL22 | NEB | SPECC1 |  | CSF3R | JAG1 | PAX3 | SF1 | ZNF292 |
| FBXO10 | NECTIN4 | SRC |  | DDIT4 | KCTD5 | PDK1 | SH3BP1 | ZNF639 |
| FOXI3 | NIPSNAP3A | SRY |  | DSCAM | KHDC4 | PDZD11 | SIGLEC7 | ZNF654 |
| FOXJ2 | NPW | SULT1D1P |  | DUSP2 | KISS1R | PEAR1 | SLC16A1 | ZNRF1 |
